# Supplementary material for: Patterns of Intron Gain and Loss in Fungi
Source: PLoS Biol. 2004 Nov 30;2(12):e422. doi: 10.1371/journal.pbio.0020422 (PMC532390; doi:10.1371/journal.pbio.0020422)
Supplement: Table S1 — Also available at http://genes.mit.edu/NielsenEtAl/. (4.3 MB ZIP). [file pbio.0020422.st001.zip › NielsenEtAl/html/852.html]

AN2875.1.NCU07807.1.MG00223.1.FG02770.1


```
 CLUSTAL W (1.82) Multiple Sequence Alignments - Introns Inserted


Sequence 1: AN2875.1	360 aa
Sequence 2: FG02770.1	360 aa
Sequence 3: MG00223.1	378 aa
Sequence 4: NCU07807.1	362 aa
Alignment Length: 380 aa
Number Identitical Residues: 226 aa
Alignment Score (without introns) 10365


MG00223.1 	MMLPVVRSIGVEPEGKEC0MGVFS~ELGLKPGVLYG~EEVYKLFEHAKKNVYAIPAI~NV
NCU07807.1	------------------~MGIFD0ELNLPAGVLYG1DDVLKLFQYAREKQFAIPAC~NV
FG02770.1 	------------------~MGVQE~VLSRKSGVIVG~DDVLRLFEYAREHKFAIPAI0NV
AN2875.1  	------------------~MGVLE~KLSRKTGVIVG~DDVLRLFEYAQEHNFAIPAI0NV
          	                   **: .  *.  .**: * ::* :**::*::: :****  **

MG00223.1 	TSSSTI2IASLEAARDSKSPIILQMSQGGAAYFAGK~GVSNTNQ--EASIAGAVAAAHFI
NCU07807.1	TSSSTA~VAALEAARDQKAPIILQTSQGGAAFFAGK~GIKDSAEKREASVAGAIAAAHYI
FG02770.1 	TSSSTV~VAALEAARDAKAPVILQFSQGGAAYFAGK0GVSNGDQ--AASIAGSVAAAHYV
AN2875.1  	TSSSTV~VACLEAARDQNCPVILQVSQGGAAFFAGK0GVSNDGQ--AASIAGGIAAAHYI
          	*****  :*.****** :.*:*** ******:**** *:.:  :   **:**.:****::

MG00223.1 	RSIAPIYGVPVVL~HTDHCAKKLLPWLDGMLDADEAFHKENGTPLFSSHMIDLSE~EPRD
NCU07807.1	RSIAPIYGIPVVL1HTDHCAKKLLPWLDGMLEEDEKFFKANGVPLFSSHMIDLSE1EPVE
FG02770.1 	RSLAPTYGIPVVL~HTDHCAKKLLPWLDGMLDADEAYFKEKGEPLFSSHMIDLSE~EPVE
AN2875.1  	RSIAPSYGIPVVL~HTDHCAKKLLPWLDGLLDADEAYFKQHGEPLFSSHMIDLSE~EPVD
          	**:** **:**** ***************:*: ** :.* :* ************ ** :

MG00223.1 	WNIETTAKYLKRAAPMKQWLEM~EIGLT~GGEED0GVNNEDVDNNSLYTQPEDIFAIHQA
NCU07807.1	ENISTCVKYLKRMAPMKQWLEM~EIGIT0GGEED~GVDNSEVDNASLYTQPEDIWQIEEA
FG02770.1 	WNIETTAKYLKRAAPMKQWLEM~EIGIT~GGEED~GVNNEDVDNNSLYTQPEDILNIYNT
AN2875.1  	YNIDTTAKYLKRAAPMKQWLEM0EIGIT~GGEED~GVNNESVDNNSLYTQPEDILAIHNA
          	 **.* .***** ********* ***:* ***** **:*..*** *********  * ::

MG00223.1 	LSPISKYFSIAAGFG~NVHGVYKPGNVRLHPELLDKHQKYVIEKLGCEEKKPI1FFVFHG
NCU07807.1	FRPISPYFSIAAGFG2NVHGVYAPGNVKLHPELLGKHQAYVSEKLGGKDKKPV~FFVFHG
FG02770.1 	LSPISPYFSIAAGFG~NVHGVYKPGNVKLHPELLGKHQAHVKEALKSDNDKPV~FFVFHG
AN2875.1  	LSKISPYFSIAAGFG~NVHGVYKPGNVRLHPELLSKHQAYVKEQTGSKKDKPV~FFVFHG
          	:  ** ********* ****** ****:******.*** :* *    ...**: ******

MG00223.1 	GSGSGDSEFQEAISYGVIKVNLDTDLQWAYLSGIRDYVTSKIEYLNSQVGNPDGADKPNK
NCU07807.1	GSGSSKEEYREAISNGVVKVNVDTDLQWSYLVGIRDYILNNIDYLRSQVGNPEGPNKPNK
FG02770.1 	GSGSSKKEYLDAIGFGVVKVNVDTDMQFAYCSGIRDYMVNKREYVNTTVGNPDGEDKPNK
AN2875.1  	GSGSSKEEYKEAISYGVVKVNLDTDMQYAYLSGVRDYVLNKKDYLMSTVGNPDGEDKPNK
          	****...*: :**. **:***:***:*::*  *:***: .: :*: : ****:* :****

MG00223.1 	KYYD~PRVWVREGEKTMKARIQQALKVFNAENTI
NCU07807.1	KKYD~PRVWIREGEKTMKARVEEALKDFNAAGTV
FG02770.1 	KYFD0PRVWVREGEKTMSKRVAEALEDFNTANQL
AN2875.1  	KFFD~PRVWIREGEKTMSKRVQVALEDFNTAGKL
          	* :* ****:*******. *:  **: **: . :
```
